# WS_2_ with Controllable Layer Number Grown Directly on W Film

**DOI:** 10.3390/nano14161356

**Published:** 2024-08-16

**Authors:** Yuxin Zhang, Shiyi Feng, Jin Guo, Rong Tao, Zhixuan Liu, Xiangyi He, Guoxia Wang, Yue Wang

**Affiliations:** School of Physical Science and Technology, Inner Mongolia University, Hohhot 010021, China

**Keywords:** tungsten disulfide, chemical vapor deposition, magnetron sputtering

## Abstract

As a layered material with single/multi-atom thickness, two-dimensional transition metal sulfide WS_2_ has attracted extensive attention in the field of science for its excellent physical, chemical, optical, and electrical properties. The photoelectric properties of WS_2_ are even more promising than graphene. However, there are many existing preparation methods for WS_2_, but few reports on its direct growth on tungsten films. Therefore, this paper studies its preparation method and proposes an innovative two-dimensional material preparation method to grow large-sized WS_2_ with higher quality on metal film. In this experiment, it was found that the reaction temperature could regulate the growth direction of WS_2_. When the temperature was below 950 °C, the film showed horizontal growth, while when the temperature was above 1000 °C, the film showed vertical growth. At the same time, through Raman and band gap measurements, it is found that the different thicknesses of precursor film will lead to a difference in the number of layers of WS_2_. The number of layers of WS_2_ can be controlled by adjusting the thickness of the precursor.

## 1. Introduction

Two-dimensional materials have attracted extensive attention due to their excellent optical [1,2,3], electrical [4,5], and electrochemical [6] properties [7,8,9,10,11]. These unique physical and chemical characteristics have not only propelled in-depth explorations in the field of materials science, but also provided strong support for the development of high-tech products such as novel electronic devices, semiconductor devices, optoelectronic devices, and sensors [12,13,14,15]. Among these two-dimensional materials, the two-dimensional transition metal sulfide WS_2_ stands out, possessing a wider bandgap and tunable bandgap properties compared to other sulfides [16,17,18]. As a type of two-dimensional graphene material, WS_2_ has a layered structure similar to that of graphene, but with unique physicochemical properties [19,20,21]. Due to its excellent electrical, optical, and electrochemical properties, WS_2_ has attracted widespread attention and has great application potential in catalysis, hydrogen storage, electronic devices, photoelectric devices, and conductor circuits [22,23,24,25,26,27,28,29,30,31,32,33,34], such as in fully photon-operated transistors/all-optical switches, neuromorphic and optical computing, hybrid nano-LED/layered chalcogenide electro-optical converters, robust switching layers for singularly addressable nano-LED lithography techniques, and many others [35,36,37,38,39,40,41,42]. Therefore, the synthesis of WS_2_ with precisely controlled size, shape, morphology, and crystal structure is of crucial importance for its applications in industry and high-tech fields [43,44,45,46].

The common preparation methods of WS_2_ include mechanical exfoliation, liquid exfoliation, hydrothermal synthesis, and chemical vapor deposition (CVD) [47,48,49,50]. For example, Huang et al. [51] obtained monolayers of WS_2_ nanosheets from bulk materials by mechanical stripping, but this method has a low yield, which makes it difficult to produce large-scale WS_2_ films. In addition, it is difficult to precisely control the size, shape, and thickness of the resulting WS_2_ nanosheets by these methods, leading to variations in the material properties, and the mechanical forces may also cause damage to the WS_2_ lattice and introduce defects during the peeling process, as well as adsorbing impurities from the environment, affecting the purity and properties of WS_2_. Lin et al. [52] prepared WS_2_ nanoribbons by hydrothermal synthesis, which is a synthetic method of chemical reaction in a high-temperature and high-pressure aqueous environment with high reactivity, but the resulting products are usually multiphase mixtures, which are difficult to isolate and purify, and there is a possibility of side reactions occurring during hydrothermal synthesis, which leads to the stability of the product structure and consistency of the particle morphology not being easy to ensure. Kun et al. [53] realized the growth of WS_2_ thin films on sapphire substrates by PLD technology. However, PLD technology is not convenient for large-area film formation, and due to the limited focal area of the laser beam, a more complex scanning system and finer control techniques are required, which increases the difficulty and cost of preparation. Chemical vapor deposition is used to deposit WS_2_ thin films on the target substrate by heating a sulfur and tungsten source directly in a CVD furnace and controlling the carrier gas flow and other conditions, but due to the limitations of one step, finer control is not achievable, which leads to the presence of defects and impurities in the film, affecting the overall quality of the film [54]. Due to these limitations, it is crucial to develop a novel method that can produce high-quality WS_2_ films with low cost, large area coverage, and controllable film thickness. This will pave the way for a general preparation route for two-dimensional materials in the future. In this paper, we propose an innovative method for growing WS_2_ films directly on metal films. Specifically, the precursor W film is first prepared by magnetron sputtering, a technique that utilizes high-energy plasma to sputter target material onto a substrate. Following this, the W film is placed in a tube furnace and subjected to chemical vapor deposition, where sulfur vapor reacts with the W film to form WS_2_. Since the two-step method separates the two processes, it can be optimized over a wider range of process parameters. To investigate the optimal conditions for WS_2_ film growth, we utilize the control variable method to study the influence of reaction temperature and precursor thickness. By adjusting these parameters, we aim to develop WS_2_ films with superior properties that are suitable for a wide range of applications.

## 2. Experiment

**Figure 1:**
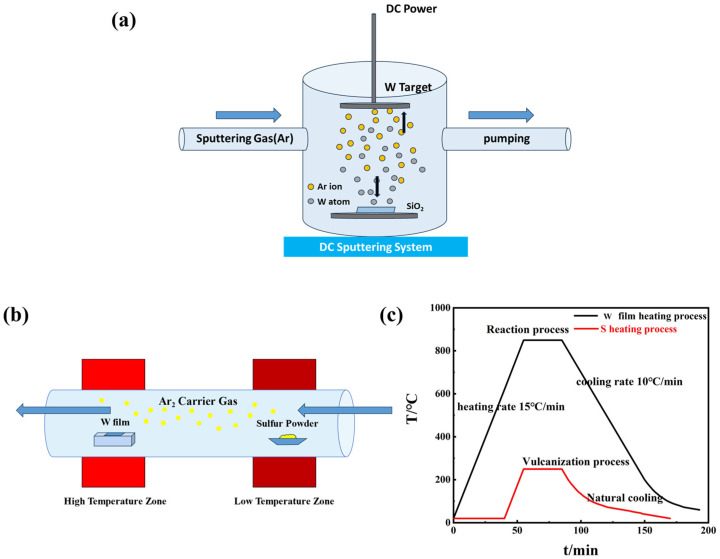
(**a**) Schematic diagram for the preparation of precursor W films by magnetron sputtering; (**b**) principle diagram of tungsten disulfide CVD growing equipment; (**c**) reaction temperature curve.

First, we used the magnetron sputtering method to deposit metal W films on a SiO_2_ substrate, after which the cavity was evacuated and then passed into argon gas. The substrate was then heated up to 100 °C and, under the action of the electric field, Ar ions accelerated to fly to the cathode W target and bombarded the target with high energy, causing sputtering of the target, so that the W atoms were deposited on the substrate to form a thin film. The reaction schematic is shown in Figure 1a. The W film was then prepared by magnetron sputtering into the quartz boat, which was placed in the middle of the high-temperature zone (800 °C–1050 °C) of the tube furnace, and the quartz boat equipped with high-purity sulfur powder was placed in the middle of the low-temperature zone at 250 °C. The schematic diagram of the reaction can be seen in Figure 1b. Before heating, the furnace should be washed two to three times, and then the system should be heated in accordance with the set temperature. The temperature should be set in accordance with the low-temperature and high-temperature area at the same time to reach the reaction temperature; the heating rate was 15 °C/min, the cooling rate was 10 °C/min, and the temperature was reduced to 200 °C for natural cooling. The temperature gradient setting curve is shown in Figure 1c.

In order to determine the crystal structure, crystallinity, and other key parameters of the crystals, and to find the distribution law of the atoms inside the crystals, the samples were subjected to X-ray diffraction (XRD) measurements, which were carried out using an X-ray diffractometer with Cu-Ka radiation; Raman testing can reveal information about the vibration and rotation energy levels of the material’s molecules, thus helping us to understand its microstructural characteristics. Meanwhile, scanning electron microscope testing provides a visual image of the material’s surface morphology, including grain size, growth direction, and possible surface defects, so combining the two testing methods allows us to gain a comprehensive and in-depth understanding of the material’s growth state. In order to measure the energy gap value of the WS_2_ material, the absorption spectrum in the UV–visible region can be analyzed by the UV-VIS (ultraviolet–visible spectroscopy) characterization technique. When the photon energy is greater than or equal to the energy gap, the electrons can be excited from the valence band to the conduction band, resulting in the absorption of light by the material.

## 3. Results and Discussion

There are many factors affecting the growth of WS_2_. Through our experiments, it is found that the sample growth under this method is sensitive to the reaction time, the gas flow rate, and the amount of S powder, and good results which have excellent performance can be obtained only under specific conditions, while the influence of the reaction temperature and precursor thickness on the growth of WS_2_ film is more valuable to report.

**Figure 2:**
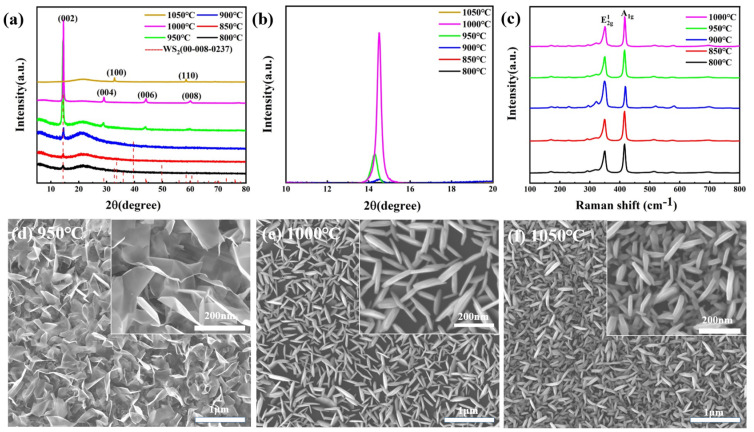
(**a**) XRD diffraction patterns at different reaction temperatures; (**b**) XRD patterns of 2θ at 10°~20°; (**c**) Raman spectra of WS_2_ grown at different temperatures; (**d**–**f**) SEM patterns of WS_2_ prepared at different reaction temperatures: (**d**) 950 °C; (**e**) 1000 °C; (**f**) 1050 °C.

The control variable method was used to study the preparation of WS_2_ by precursor W film at different reaction temperatures (800, 850, 900, 950, 1000, and 1050 °C). Figure 2a shows the XRD diffraction pattern of WS_2_ prepared at different reaction temperatures, in which the 2θ interval is 5°~80°. It can be seen from the figure that within the reaction temperature range of 800~1000 °C, the characteristic peaks are located at 2θ = 14.363°, 28.858°, 44.054°, and 60.008°. They belong to (002), (004), (006), and (008) crystal planes, respectively. These peaks all belong to the same direction perpendicular to the WS_2_ laminae and are uniformly spaced, indicating that we have grown small single-crystal WS_2_ with the same orientation and neatly aligned interlayers. WS_2_ crystals have the lowest (002) surface energy, so they all grow in the (002) direction. When the temperature rose to 1050 °C, it was found that the position of characteristic peaks changed, and crystal planes (100) and (110) (which were not found at other temperatures) appeared. This is thought to be caused by the exposure of the metal layer to sulfur vapor at high temperatures, a reaction that not only alters the internal structure of the metal layer but also contributes to the creation of disordered polycrystalline films. Figure 2b shows the local XRD pattern of the 2θ interval of 10° to 20°. There is almost no peak intensity at 800 and 850 °C, indicating that the crystallinity is not high at this time. With the increase in temperature, the peak has an obvious bulge and reaches its peak at 1000 °C, when the peak width is also the narrowest, indicating that with the increase in temperature, the crystallinity of the crystal is also continuously improved. This is due to the fact that the increase in temperature causes faster evaporation of the precursor film, allowing for a fuller reaction with the sulfur vapor. These findings illustrate that 1000 degrees Celsius is a good sample preparation condition.

Raman spectroscopy and scanning electron microscopy analysis were carried out in order to probe deeply into the microstructural characteristics of the materials and their crystallization states during growth. Figure 2c shows the Raman spectra of WS_2_ at different temperatures. The characteristic vibration modes of WS_2_, $E_{2g}^{1}$ and $A_{1g}$ are clearly presented on the measurement plots of all samples, further indicating that we have grown better-quality WS_2_. Figure 2d–f show the SEM images of WS_2_ grown at different temperatures. When the temperature reaches 950 °C, a combination of rods and flakes can be seen. When it reaches 1000 °C, all the flake structures are formed, and they are perpendicular to the base. The transverse size of each flake is about 30 nm. When the temperature was further raised to 1050 °C, a rod-like structure was grown. The reason for the change in sample topography should be temperature. We speculate that, when the temperature is low, the sample grows horizontally as a sheet, and when the temperature is high, the sample rises upright off the surface, and when the temperature is even higher, the sample rolls up and forms a rod-like structure. The crystal direction at 1000 °C is different from other temperatures in XRD, which indicates vertical growth. Due to its vertical growth, the contact surface becomes larger, which is very conducive to catalytic hydrogen evolution reactions.

**Figure 3:**
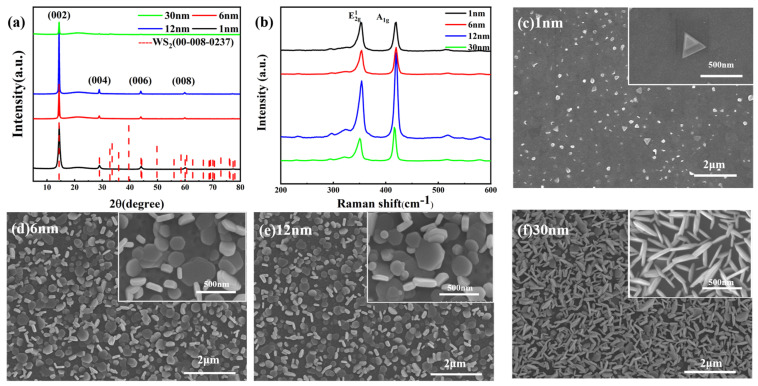
(**a**) XRD diffraction patterns of WS_2_ prepared from different thicknesses of precursor W films; (**b**) Raman patterns of WS_2_ prepared from different thicknesses of precursor W films; (**c**~**f**) SEM patterns of WS_2_ prepared from different precursor thicknesses: (**c**) 1 nm; (**d**) 6 nm; (**e**) 12 nm; (**f**) 30 nm.

Another growth parameter with a particular effect is the thickness of the precursor. In this experiment, the thickness of the precursor W film was set as 1 nm, 6 nm, 12 nm, and 30 nm under the control of the reaction temperature, reaction time, Ar flow rate, and S powder quantity. Figure 3a shows the XRD patterns of WS_2_ grown with different precursor thicknesses. We see from Figure 3a that the 1 nm~30 nm precursor W film growing WS_2_ in 2θ = 14.3°, 28.8°, 43.9°, and 59.81° formed a WS_2_ six-party structure, with four peaks, respectively, belonging to the (002), (004), (006), and (008) crystal planes. High-intensity (002) peaks indicate that grains with the (001) direction are grown. Figure 3b shows the Raman spectrum of WS_2_ prepared with a 1 nm~30 nm W membrane as a precursor, and the two vibration modes of WS_2_ ($E_{2g}^{1}$ and $A_{1g}$) are well displayed. The two peaks of $E_{2g}^{1}$ and $A_{1g}$ are located near 353 cm^−1^ and 419 cm^−1^, respectively. They represent in-plane and out-of-plane vibrations, and the interval between the two vibration modes is closely related to the thickness of the film. The thickness-dependent interlayer coupling effect indicates that the frequency difference varies with the precursor thickness, which indicates that different numbers of WS_2_ layers are generated. The frequency differences between the two main vibrational modes for different precursor thicknesses are shown in Table 1. Specifically, with the increase in precursor thickness, the number of WS_2_ layers increases and the interlayer interaction is enhanced, leading to a decrease in the frequency difference. This finding provides an experimental basis for the precise control of the number of WS_2_ layers by modulating the precursor thickness, which is of great significance for the optimization of the material properties and its application in electronics and optoelectronics. 

In order to further observe whether the number of sample layers changed, we performed SEM measurements to visualize the evolution of the film morphology of the precursor under different thickness conditions. Figure 3c–f show the SEM images of precursors at 1 nm~30 nm, respectively, and the inset shows the local magnified images. It can be seen that, when the precursor film is 1 nm, as shown in Figure 3c, triangles grow inside the film and tiny triangular structures grow on the surface of the film, and these triangles are of different sizes, with the largest triangle having a size of about 1 μm. Based on the previous studies, it is very likely that these triangular WS_2_ nanostructures contain only a single layer or a few layers, as their morphological features coincide with the known properties; triangular WS_2_ tends to have only one or a few layers. As shown in Figure 3d,e, when the thickness of the precursor is increased to 6 nm and 12 nm, most of the thin-film nuclei exhibit a pentagonal shape, with some lamellae inserted perpendicularly into the substrate and some of the lamellae parallel to the substrate. This morphological transition indicates an increase in the number of WS_2_ layers, and according to previous studies, pentagonal WS_2_ tends to have more layers. Further WS_2_ grown with a 12 nm precursor is thicker than WS_2_ grown with a 6 nm precursor, indicating a higher number of layers. When the precursor thickness increases to 30 nm, as shown in Figure 3f, the samples become rod-like, implying a large number of layer stacks, indicating that the number of layers is already large. Therefore, the scanning electron microscopy results indicate that the number of WS_2_ layers increases as the precursor thickness increases. Through the results of the scanning electron microscopy measurements, we clearly observed and confirmed the phenomenon that the number of WS_2_ layers gradually increased with the increase in the precursor thickness, which deepened our understanding of the growth mechanism of WS_2_ films.

**Figure 4:**
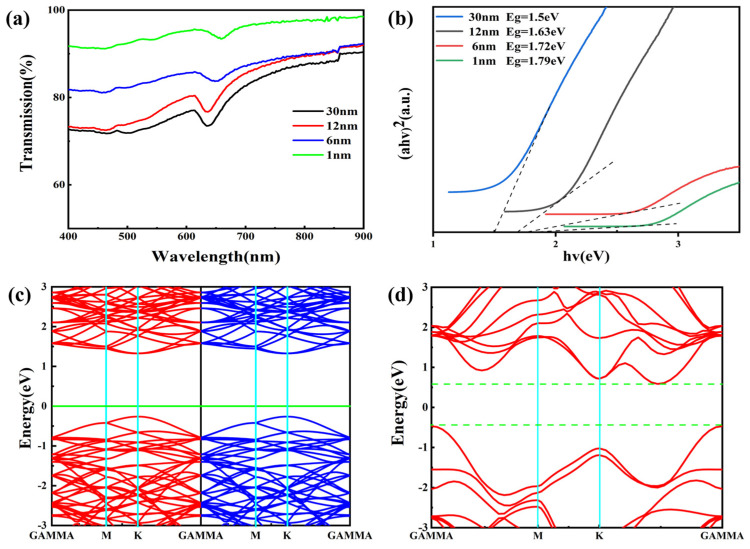
(**a**) Transmission spectra of WS_2_ films as a function of W-film thickness; (**b**) determination of the band gap of WS_2_ prepared with different precursor thicknesses using the Tacu method; (**c**) single-layer WS_2_ energy band diagram; (**d**) multilayer WS_2_ energy band diagram.

The WS_2_ energy gap of different layers is different, so the number of layers can also be characterized by measuring the size of the energy gap. The band gap of layered WS_2_ prepared by W films with different precursor thicknesses was obtained by using UV-VIS spectroscopy, and their optical transmittance is shown in Figure 4a. The wavelength range was set between 400 nm and 900 nm. The transmittance of the generated WS_2_ films decreases gradually at precursor W-film thicknesses of 1 nm, 6 nm, 12 nm, and 30 nm, which are 93.4%, 83.98%, 75.56%, and 73.83%, respectively. This reduction in transmittance indicates an increase in the number of layers of the sample, since thicker WS_2_ films mean that more light is absorbed or scattered, which reduces the transmittance. To further quantify the energy gap values, Figure 4b shows the relationship between (αhv)^2^ and hv obtained by the Tacu method. As the photon energy increases, the absorption edge of each sample is gradually revealed, and the intercept between the tangent of the absorption edge and the X-axis corresponds to the respective band gap value. It can be seen that the band gaps of the corresponding WS_2_ films are 1.5 eV, 1.6 eV, 1.72 eV, and 1.79 eV when the thickness of the precursor W films is 1 nm, 6 nm, 12 nm, and 30 nm, respectively. 

Meanwhile, we describe the interactions between valence electrons and nuclei by the projective affix plus plane wave PAW method based on the density-functional theory (DFT) using the VASP package, and the exchange-correlation effects between valence electrons are described by the generalized gradient approximation (GGA) and PBE. A WS_2_ supercell structure of 4 × 4 × 1 with a total of 48 atoms is selected as the object of study for the computational system, and a 9 × 9 × 1 K-point lattice is generated by the Monkhorst–Pack scheme to optimize the geometry and relax the atomic positions until the force acting on each ion is less than 0.05 eV/Å. The difference in energy between adjacent iterations is less than 1 × 10^−6^ eV when the relaxation is stopped, and a kinetic cutoff energy of 400 eV is set for the plane-wave-basis group. A vacuum layer of 20 Å in the c-axis direction is used to prevent layer-to-layer interactions during modeling. Energy band calculations were performed for single-layer WS_2_ and multilayer WS_2_, as shown in Figure 4c,d, where the left side of the energy band of single-layer WS_2_ indicates spin-up and the right side indicates spin-down, while the minima of the conduction band and the maxima of the valence band are in the same wavevector, K. Therefore, the single-layer WS_2_ is a direct bandgap with the computed bandgap of 1.813 eV, whereas the multilayer WS_2_ is an indirect bandgap with a bandgap of 1.2 eV. In turn, this indicates that the bandgap gradually increases as the number of layers decreases and the bandgap gradually changes from an indirect bandgap to a direct bandgap. The band gap obtained by our experiment increases in turn with the decrease in the film thickness of the precursor, indicating that the number of WS_2_ layers we grow is decreasing [55].

From all the above experimental results, we conclude that as the thickness decreases, the number of WS_2_ layers also decreases. That is to say, the number of WS_2_ layers can be regulated by controlling the thickness of precursor films. WS_2_ with different numbers of layers has different photoelectric performance. For example, the energy band structure of monolayer WS_2_ is able to change from an indirect band gap to a direct band gap; the direct-bandgap WS_2_ performs better in light absorption and light emission and can utilize light energy more efficiently, thus improving the photoelectric conversion efficiency of the device. The controllable number of layers will promote the further development of WS_2_ in the application of photoelectric devices.

Although this study verified the effectiveness and feasibility of the proposed method, its future scale-up of the process in industrial applications may face a series of challenges, including, but not limited to, the stability of the raw material supply, the compatibility and stability of the equipment, the control of the production cost, and the assessment of the environmental impact. These challenges are the directions that future research should focus on to ensure that the method can be successfully applied in real industrial production.

## 4. Conclusions

In this paper, a new method for direct growth of stable large-area WS_2_ on tungsten films using a double-temperature tube furnace has been proposed. This approach not only overcomes many of the shortcomings of the conventional growth process, but also provides a new perspective to understand and control the growth process of two-dimensional materials. At the same time, we found that the horizontal or vertical growth of WS_2_ could be controlled by changing the growth temperature. Vertically growing WS_2_ has great potential in terms of catalytic performance due to its large contact area. This vertically grown WS_2_ not only provides more active sites but also enhances interfacial interactions with other materials, thus exhibiting higher efficiency and selectivity in catalytic reactions. In addition, the number of WS_2_ layers growing can be controlled by changing the thickness of the precursor tungsten film. This discovery provides us with another way to modulate the performance of WS_2_. By precisely controlling the number of layers of WS_2_, we can achieve the customization of its electrical and optical properties, thus promoting the development of WS_2_ applications in optoelectronic devices, sensors, and other fields.

## Figures and Tables

**Table 1:** The frequency difference between the two main vibrational modes of precursors with different thicknesses.

Precursor Thickness (nm)	Frequency Difference (cm^−1^)
1	67.542
6	67.057
12	66.582
30	65.794

## Data Availability

Data is contained within the article.
